# Identification of germline alterations of the mad homology 2 domain of SMAD3 and SMAD4 from the Ontario site of the breast cancer family registry (CFR)

**DOI:** 10.1186/bcr2926

**Published:** 2011-08-11

**Authors:** Eric Tram, Irada Ibrahim-zada, Laurent Briollais, Julia A Knight, Irene L Andrulis, Hilmi Ozcelik

**Affiliations:** 1 Fred A. Litwin Centre for Cancer Genetics, Samuel Lunenfeld Research Institute, Mount Sinai Hospital, Department of Laboratory Medicine and Pathobiology, University of Toronto, 60 Murray St., Toronto, ON M5T 3L9, Canada; 2 Prosserman Centre for Health Research, Samuel Lunenfeld Research Institute, Mount Sinai Hospital, Dalla Lana School of Public Health, University of Toronto, 600 University Avenue, Toronto, ON M5G 1X5, Canada; 3 Cancer Care Ontario, Samuel Lunenfeld Research Institute, Mount Sinai Hospital, Department of Molecular Genetics, University of Toronto, 60 Murray Street, Toronto, ON M5T 3L9, Canada

## Abstract

**Introduction:**

A common feature of neoplastic cells is that mutations in SMADs can contribute to the loss of sensitivity to the anti-tumor effects of transforming growth factor-β (TGF-β). However, germline mutation analysis of *SMAD3 *and *SMAD4*, the principle substrates of the TGF-β signaling pathway, has not yet been conducted in breast cancer. Thus, it is currently unknown whether germline *SMAD3 *and *SMAD4 *mutations are involved in breast cancer predisposition.

**Methods:**

We performed mutation analysis of the highly conserved mad-homology 2 (MH2) domains for both genes in genomic DNA from 408 non-*BRCA1/BRCA2 *breast cancer cases and 710 population controls recruited by the Ontario site of the breast cancer family registry (CFR) using denaturing high-performance liquid chromatography (DHPLC) and direct DNA sequencing. The results were interpreted in several ways. First, we adapted nucleotide diversity analysis to quantitatively assess whether the frequency of alterations differ between the two genes. Next, *in silico *tools were used to predict variants' effect on domain function and mRNA splicing. Finally, 37 cases or controls harboring alterations were tested for aberrant splicing using reverse-transcription polymerase chain reaction (PCR) and real-time PCR statistical comparison of germline expressions by non-parametric Mann-Whitney test of independent samples.

**Results:**

We identified 27 variants including 2 novel *SMAD4 *coding variants c.1350G > A (p.Gln450Gln), and c.1701A > G (p.Ile525Val). There were no inactivating mutations even though c.1350G > A was predicted to affect exonic splicing enhancers. However, several additional findings were of note: 1) nucleotide diversity estimate for *SMAD3 *but not *SMAD4 *indicated that coding variants of the MH2 domain were more infrequent than expected; 2) in breast cancer cases *SMAD3 *was significantly over-expressed relative to controls (*P *< 0.05) while the case harboring *SMAD4 *c.1350G > A was associated with elevated germline expression (> 5-fold); 3) separate analysis using tissue expression data showed statistically significant over-expression of SMAD3 and SMAD4 in breast carcinomas.

**Conclusions:**

This study shows that inactivating germline alterations in *SMAD*3 and *SMAD*4 are rare, suggesting a limited role in driving tumorigenesis. Nevertheless, aberrant germline expressions of *SMAD3 *and *SMAD4 *may be more common in breast cancer than previously suspected and offer novel insight into their roles in predisposition and/or progression of breast cancer.

## Introduction

The *BRCA1 *and *BRCA2 *tumor suppressor genes have been established as important high penetrance familial breast cancer susceptibility alleles [1]. Rare mutations of other tumor suppressor genes involved in direct protein-protein interaction with *BRCA1/2 *including *TP53, PTEN, CHEK2, ATM, NBS1, RAD50, BRIP1*, and *PALB2 *were also discovered in breast cancer families, altogether accounting for up to 50% of familial breast cancers [2,3]. On the other hand, rare germline alterations of potential disease genes have not been investigated for the most common non-familial (sporadic) form of breast cancer, which accounts for the majority (70 to 80%) of all breast cancers in the population.

Tumor suppressor genes known to be somatically inactivated in breast cancers are particularly attractive candidates. SMAD3 and SMAD4 are the key signaling proteins of the transforming growth factor-β (TGF-β) pathway and have been implicated to have tumor suppressive effects in the pathogenesis of breast and other cancer types [4,5]. The binding of TGF-β to TGFBI and TGFBII receptors results in the activation of SMAD2/3 and hetero-complex formation with SMAD4 [6] and mediates the regulation of genes involved in the suppression of epithelial cell growth following nuclear translocation. SMAD3 and SMAD4 possess two evolutionarily conserved domains termed Mad-homology 1 (MH1) and 2 (MH2). The N-terminus MH1 domain is a DNA-binding domain recognizing CAGA motifs. The C-terminus MH2 domain is highly conserved and is one of signal transduction's most versatile protein-interacting domain. It is involved in the interaction with TGFBR1, formation of SMAD homomeric or heteromeric complexes, and transcriptional activation (Reviewed in [7]).

The loss of SMAD3 expression and function is involved in susceptibility to gastric cancers, colorectal cancers and acute T-cell lymphoblastic leukemia [8-10]. Several lines of evidence suggest that *SMAD3 *may be involved in breast cancer susceptibility. The *SMAD3 *locus on chromosome 15q21 has been shown to undergo allelic imbalance [11]. In addition, SMAD3, like many breast cancer susceptibility genes, is in direct protein-protein interaction with BRCA1 as it counteracts BRCA1-mediated DNA repair [12] and its MH2 domain has recently been shown to associate with BRCA1 during oxidative stress response [13]. While inactivating mutations in *SMAD3 *were previously believed to be absent in all cancer types [14], a putative inactivating missense mutation (R373H) was found in the colorectal cancer cell line SNU-769A [15] as well as c.1009+1G > A and c.1178C > T (P393L) from the screening of 38 primary colorectal cancers [16] both localized to the MH2 domain.

*SMAD4/DPC4 *is a tumor suppressor gene, which is mutated or deleted in half of all human pancreatic carcinomas [17] and loss of expression (LOH) has been shown to be important for the progression of gastric [18], cervical [19] and colorectal [20] cancers. At least 15% of breast tumors exhibit LOH at the 18q21 locus on which *SMAD4 *is situated [21] and breakpoints in this region are associated with minimum copy number [22] suggesting a tumor suppressor role. In addition to pancreatic cancer, *SMAD4 *is somatically inactivated in colon and biliary cancers [23], gastric cancer [24], homozygous deletions of *SMAD4 *have been detected in a small percentage of invasive ductal carcinomas [25,26]. In the germline, inactivating *SMAD4 *mutations are found to be associated with approximately 20% of Juvenile Polypopsis Syndrome (JPS) cases [27,28]. Consequently, mutation analyses in many cancers have highlighted the MH2 domain of SMAD4 as a mutational hotspot [29].

Presently, it is not known whether *SMAD3 *and *SMAD4 *germline alterations are involved in breast cancer predisposition. Here, we aimed to explore the mutation spectrum of *SMAD3 *and *SMAD4 *by screening the highly conserved MH2 domain in the germline DNA in familial and non-familial breast cancer cases as well as age, gender and ethnicity matched healthy population controls.

## Materials and methods

### Study population

Although considered different, familial and sporadic forms of breast cancers have been shown to have common biological mechanisms, affecting similar pathways such as alterations to BRCA-associated function in both forms [30]. For example, a considerable portion of patients with triple negative breast cancers (that is, those that do not express estrogen receptor (ER), progesterone receptor (PR), and the human epidermal growth factor receptor HER2) might also carry *BRCA1/2 *mutations [31-33]. Additionally, a fraction of the breast cancers may be misclassified based on the truncated family history; therefore, making a fuzzy line between familial and sporadic cases. To represent a breast cancer population sample that is not only sporadic or familial, we took advantage of the population-based sample of the Ontario Familial Breast Cancer Registry (OFBCR), a participating site in the US NIH Breast Cancer Family Registry (BCFR) [34,35].

Familial cases of OFBCR were sampled from probands that met certain high-risk criteria, including having at least one first-degree relative with breast and/or ovarian cancer, with second degree relatives with breast and ovarian cancer, or with additional cancers (for example, prostate, pancreatic, and so on) in the first or second degree relatives and who was diagnosed at age < 36 with multiple breast and/or ovarian primaries, or Ashkenazi Jewish background. The breast cancer cases that did not meet the familial criteria listed above were classified as non-familial (sporadic) breast cancer cases, which are represented by the older patients with no family history of breast cancer. All the familial breast cancer cases included in this study were previously tested negative for *BRCA1/2 *mutations. The age range of all participating women was 25 to 69 years, with an average age of 48.8 ± 9.26 years. Female non-cancer population controls have been randomly identified using the listed, residential telephone numbers for the province of Ontario. Controls were frequency-matched to female case probands based on their expected five-year age distribution and ethnicity (64% response rate). The registry sample consists of about 90% Caucasian women and healthy female population controls with the reference age in the range of 23 to 69 with an average age of 49.1 ± 9.55 years. Written informed consent was obtained from all subjects, and Mount Sinai Hospital Research Ethics Board approved the study protocol. Genomic DNA was extracted from blood lymphocytes from a total of 408 breast cancer (173 familial and 235 sporadic) and 710 non-cancer population controls (20% (141/710) sharing a familial criteria) were randomly selected and subjected to genetic analysis.

### Genetic analysis

Polymerase chain reaction (PCR) was used to amplify the exons and exon-intron boundaries of exons 7, 8, 9 and exons 8, 9, 10, 11 spanning the MH2 domains of *SMAD3 *and *SMAD4*, respectively. Thermocycling conditions and PCR primer sequences are summarized in Table 1. PCR was carried out in 50 μl volume containing 10 ng of genomic DNA, 1xPCR Gold buffer, 25 ng of each 10 mmol/l primer, 2.5 units of Taq DNA polymerase (AmplitaqGold; Perkin-Elmer, Branchbury, NJ, USA). Thermocycling was carried out in a Bio-Rad Dyad thermocycler (Mississauga, Ontario, Canada) and evaluated on 1.5% agarose gels. To ensure proper formation of homo- and hetero-duplexes for subsequent dHPLC analysis, PCR products were denatured again at 95°C for 3 minutes and re-natured for 30 minutes by decreasing temperature from 95°C to 65°C.

**Table 1:** PCR and dHPLC conditions

						PCR		DHPLC
Gene	Exon	Domain	Forward PCR Primer (5'to 3')	Reverse PCR Primer (5' to 3')	Amplicon Size (bp)	Annealing Temperature (°C)	MgCl (nM)	Melting Temperatures (°C)
SMAD3	7	MH2	CGGCAGTGCCCATTTCCCCTAC	CTAATCCAATCACCTCCAGATT	450	60	3	60, 62.5
SMAD3	8	MH2	TATAAATGAGGCTGGTCTAGGG	GACATGCCTACTACGACCGTAG	544	60	2	60.2, 62.2
SMAD3	9	MH2	GTTTAACTCTTTAAAGTCGACT	ACAGCTGTTCATAACATCCACC	556	60	2	58
SMAD4	8	MH2	TTTAAGAACAGTGCTAAGTACT	TTAAGATGGAGTGCTTACAAAT	566	60	4	51.5, 53.5
SMAD4	9	MH2	TTTAATTTTTCAATATTAAGCA	TAGATTACTGATAATGTCAATA	411	54	4	51.5
SMAD4	10	MH2	TAATGAAACTGAGTTTTAAATAA	ATTTTACCAATTCAAAAATGTCA	377	57	3	51, 53
SMAD4	11	MH2	CTTTAGCAGAGAAGTTATATGCT	AATATATCTTCAGATTATAAACA	424	57	4	59

Polymerase Chain Reaction (PCR) cycle conditions: four minutes 94°C initial denaturation; 94°C for 30 seconds, 0.5 to 1 minute at specified temperature (Ta), 72°C for 0.5 to 1 minute for 35 cycles; and a final extension step of seven minutes at 72°C. Where applicable, Denaturing High-Performance Liquid Chromatography (DHPLC) was run at two melting temperatures to obtain optimal separation.

The PCR amplicons were screened by denaturing High-Performance Liquid Chromatography (dHPLC). The optimal melting temperature was calculated using the dHPLC Melt Program [36] and DNA from breast cancer cell lines (MDA-MB453, MDA-MB468, TD47) was used to optimize the running conditions to enhance mutation detection sensitivity on the Transgenomic WAVE 4500HT (Transgenomic Inc., Omaha., NE, USA). Approximately 10 ng of DNA from cases and population controls were analyzed. Samples with elution profiles characteristic of hetero-duplexes were identified using the Navigator 1.7.0 Software (Transgenomic Inc., Omaha., NE, USA). As an internal control, a fraction of case and control samples were duplicated across our study population to ensure accuracy of the results. All samples with heteroduplex profiles were purified by SAP/ExoI and direct sequencing was performed by The Centre for Applied Genomics, The Hospital for Sick Children, Toronto, Canada.

### Nucleotide diversity

Nucleotide diversity and its standard deviation were calculated under the assumptions of an infinite site neutral allele model:

$$\theta =k∕aL,\;\;s\left(\theta \right)=\sqrt{a\theta L+b{\left(\theta L\right)}^{2}∕aL}\;a=\overset{n}{\underset{i=2}{\sum }}\frac{1}{\left(i-1\right)}\;b=\overset{n}{\underset{i=2}{\sum }}\frac{1}{{\left(i-1\right)}^{2}}$$

where K is the number of SNPs identified in a genomic length, L base pairs and n is the number of alleles analyzed.

### *In silico *analyses

Impact of missense variants on protein function was assessed by evolutionary conservation analysis using SIFT [37] and structure by PolyPhen [38] FastSNP [39] was used to evaluate the effect of synonymous variants on Exonic Splicing Enhancers (ESE) in alternative splicing regulation. The effect of intronic variants on the consensus donor site, acceptor site, branch point as well as creation of cryptic sites were carried out by measuring 5' and 3' splice site scores using Automated Splice Site Analyses (ASSA) [40]. ASSA has been shown to be as robust [41] as other prevalent splice predictors NNSplice, SpliceSiteFinder, and MaxEntScan. All *in silico *splicing analysis tools were run at default threshold values and the outputs for wildtype versus variant were documented.

### Analysis of aberrantly spliced transcripts

A total of 37 mRNA samples, 18 for SMAD3 and 19 for SMAD4, were extracted from the lymphocytes of cases and controls harboring the rare genetic variants (defined as < 5 times) identified in this study. Reverse-transcription PCR (RT-PCR) primers targeting the flanking exons of the MH2 domain of SMAD3 (exons 6, 9) and SMAD4 (exons 8, 11) based on cDNA sequences [GenBank:NM_5902 and GenBank:NM_005359, respectively]. This assay was carried out using instructions provided by SuperScript III One-Step RT-PCR System with Platinum *Taq *DNA Polymerase kit (Invitrogen Burlington, Ontario, Canada). Conditions and primer sequences are summarized in Additional file 1, Table S1. The RT-PCR products were separated on a 1.5% agarose and a non-denaturing 8% polyacrylamide gel (29:1) to ensure high resolution of fragments, and sequence was confirmed by direct sequencing of the gel-purified DNA.

### Analysis of mRNA expression levels

Quantitative Real-time PCR (qPCR) was performed using an ABI 7700 Sequence Detection System (PE Applied-Biosystems Streetsville, Ontario, Canada) in the presence of SYBR-green in a 30 μl reaction. The SYBR-Green I core reagent protocol was followed and all reagents were provided in the core reagent kit. PrimerBank [42] qPCR primers for SMAD3 (PrimerBank ID 5174513a2) and SMAD4 (PrimerBank ID 4885457a2) were used. All reactions were run in triplicates and incubated in a 96-well optical plate at 95°C for 10 minutes, followed by 40 cycles of 95°C for 15 s and 60°C for 10 minutes. Standard curves were generated using 10-fold dilutions of pooled cell-line cDNA. β-actin (Forward5'ATCATGTTTGAGACCTTCAA3', Reverse 5 CATCTCTTGCTCGAAGTCCA3') was chosen as a standard reference gene for the assay for normalization.

### mRNA expression analysis in normal breast and carcinoma tissues

The cDNA expression data from breast tumor tissues of 50 patients with invasive ductal carcinoma (IDC) and 10 samples of normal breast tissue taken from surrounding healthy breast tissue of cancer patients [43] were downloaded from ArrayExpress (accession number: E-TABM-276). Five probes (239448_at, 218284_at, 205396_at, s05397x_at, s05398_s_at) for *SMAD3 *and two (202527_s_at, 1563703_at) for *SMAD4 *from the AffymetricGeneChipU133 Plus 2.0 arrays were available.

### Identification of somatic mutations in cancer

The COSMIC database v44 release is a project that catalogues homozygous or heterozygous somatic missense mutations and deletions in various cancer types based on curated research publications [44]. Using this resource we reviewed the number of currently known somatic mutations of *SMAD3 *and *SMAD4 *in breast, colorectal and pancreatic cancers.

### Statistical analysis

Statistical analysis of germline expression with t-test, and non-parametric Mann-Whitney test of independent samples were performed using SPSS v.13.0 (SPSS Inc, Chicago, IL, USA) Statistical significance was assumed at *P *< 0.05. Fold change in tumor versus normal tissue expression was determined by two independent samples t-test and Levin's test for the equality of variance on the mean expression levels.

## Results

### *SMAD3 *and *SMAD4 *germline alterations are primarily intronic

We identified a total of 11 and 16 distinct genetic variants in the MH2 domains of *SMAD3 *and *SMAD4*, respectively (Table 2). *SMAD3 *variants were detected in 0.25% (1/408) of cases, 0.98% (7/710) of controls and 0.27% (3/1,118) in cases and controls and all were intronic variants in the form of single base substitutions or small deletions. The frequency of variants in *SMAD4 *was found to be 0.98% (4/408) in only cases and 1.13% (8/710) in only controls, and 0.36% (4/1,118) present in both cases and controls. Among these were three coding variants in *SMAD4 *including c.1214T > C (p.Phe362Phe; rs1801250) found in a case and control, and two novel variants c.1350G > A (p.Gln450Gln) and c.1701A > G (p.Ile525Val) from a case and control, respectively (Table 2).

**Table 2:** Germline variants detected in SMAD3 and SMAD4 in breast cancer

Gene	Exon	Variants	# Times (%)	RefSnp (rs) number	Case/Control	*In silico *Prediction
SMAD3	9	IVS9+132A > T	1 (0.25%)	Novel	Case	No effect
SMAD3	7	IVS6-132 C > T	1 (0.18%)	Novel	Control	No effect
	7	IVS7+69 G > C	4 (0.72%)	rs58056680	Control	No effect
	8	IVS8-48 T > G	4 (0.72%)	Novel	Control	No effect
	8	IVS8+161 C > T	1 (0.18%)	Novel	Control	No effect
	9	IVS8-211 C > T	1 (0.18%)	rs56264428	Control	No effect
	9	IVS8-170 C > T	1 (0.18%)	Novel	Control	No effect
	9	IVS8-55 A > G	2 (0.36%)	rs28410524	Control	Abolish branch site
SMAD3	7	IVS6-113 G > T	7 (1.72%); 15 (2.11%)	rs2289791	Case & Control	No effect
	7	IVS6-95 T > C	4 (0.98%); 15 (2.11%)	rs2289790	Case & Control	No effect
	8	IVS8+23 A > C	2 (0.49%); 5 (0.70%)	rs55678244	Case & Control	No effect
SMAD4	9	IVS9+118A > G	1 (0.25%)	Novel	Case	No effect
	10	IVS10+41G > A	1 (0.25%)	Novel	Case	No effect
	10	c.1350G > A/p.Gln450Gln	1 (0.25%)	Novel	Case	Loss of ESE motifs
	11	IVS10-33T > A	1 (0.25%)	Novel	Case	Cryptic branch site
SMAD4	8	IVS7-121 A > C	1 (0.18%)	Novel	Control	No effect
	8	IVS8+44 T > C	1 (0.18%)	rs28539779	Control	No effect
	9	IVS9+43delTT	1 (0.18%)	Novel	Control	No effect
	9	IVS9+68delGAA	1 (0.18%)	Novel	Control	No effect
	9	IVS9+126 del7	1 (0.18%)	Novel	Control	Cryptic branch site
SMAD4	11	IVS10-52 A > T	1 (0.18%)	Novel	Control	Cryptic donor
	11	c.1701A > G/p.Ile525Val	1 (0.18%)	Novel	Control	No effect
	11	IVS11+53 A > G	1 (0.18%)	Novel	Control	No effect
SMAD4	8	c.1214T > C/p.Phe362Phe	1 (0.25%); 1 (0.18%)	rs1801250	Case & Control	Loss of ESE motifs
	8	IVS8+109 A > G	1 (0.25%); 3 (0.42%)	Novel	Case & Control	Cryptic donor
	10	IVS10+132delA	3 (0.74%); 2 (0.28%)	Novel	Case & Control	No effect
	11	IVS11+11 C > T	8 (1.96%); 11 (1.55%)	rs1163402	Case & Control	No effect

**Note: Variant description is based on HGVS nomenclature**. Nucleotide positions were numbered based on genomic sequences of SMAD3 [GenBank:NC_000015.8] and SMAD4 [GenBank:NC_000018.8]. The cDNA sequences referenced were based on nucleic acid sequences for SMAD3 [GenBank:NM_5902] and SMAD4 [GenBank:NM_005359].

### Nucleotide diversity estimation

Under the neutral theory of molecular evolution and infinite sites model, sequence diversity can be estimated by the heterozygosity per nucleotide site (π), termed nucleotide diversity, or by the mutation parameter (θ). Both are correct for sample size and the length of region screened [45,46] and are nearly equivalent. We reported θ and compared our data to other gene sets including the coding and adjacent non-coding regions of 106 genes from clinically relevant pathways including cardiovascular, neuropsychiatry, endocrinology in 57 individuals by Cargill *et al*. [47], the 5' and 3' UTR, intron, and coding region of 75 candidate genes involved in blood pressure homeostasis in 74 individuals by Halushka *et al*. [48], and the rate of polymorphisms in the coding regions and 3' UTR of highly conserved and essential genes involved in DNA replication and transcription by Ten Asbroek *et al*. [49] (Additional file 1, Table S2a, b). Results reported for the European/American ethnic subgroups were used where applicable.

The frequency of coding variants of *SMAD3 *in cases and controls (θ = 0) was significantly lower compared to *SMAD4 *(θ = 3.99 × 10^-4 ^and 3.71 × 10^-4^, respectively) (Table 3) and lower than expected when compared to rates observed by Cargill *et al*., (θ = 5.43 × 10^-4^), Halushka *et al*., (θ = 4.5 × 10^-4^) and Ten Asbroek *et al*. (θ = 2.00 × 10^-4^). For *SMAD4*, the θ values for coding variants in cases and controls (θ = 3.99 × 10^-4 ^and 3.71 × 10^-4^, respectively) is comparable to that reported by Cargill *et al*., (θ = 5.43 × 10^-4^) and Halushka *et al*., (4.5 × 10^-4^), with standard deviations overlapping, indicating that *SMAD4 *is not preferentially altered in the germline for breast cancer.

**Table 3:** Nucleotide diversity (*θ *× 10^-4^)

**Nucleotide Diversity (θ) × 10^-4 ^(Std Dev)**							
	SMAD3		SMAD4		Cargill *et al*. [44]	Halushka *et al*. [45]	Ten Asbroek *et al*. [46]
	
	Cases	Controls	Cases	Controls			
Non-coding	13.24 ± 7.02	11.24 ± 4.00	7.56 ± 3.36	11.71 ± 4.17	5.3 ± 1.33	5.4 ± 1.5 *	5.7 ± 1.9 **
Coding	0	0	3.99 ± 2.91	3.71 ± 2.69	5.43 ± 1.36	4.5 ± 1.2 *	2.00 ± 0.61
Total	3.54 ± 1.88	8.23 ± 2.93	6.18 ± 2.44	8.61 ± 2.86	5.39 ± 1.36	8.27 ± 1.9	2.00 ± 0.61

Note: Values are mean ± SD. Due to the fact that the Halushka *et al*. study was split into half African ethnicity and half north American of European descent, only the coding/non-coding data from the European/American samples subset (*) are used for the purpose of this comparison. Please note we used θ in 3' UTR for non-coding data for Ten Asbroek *et al*. study (**) as they did not perform any analysis on intronic sequences.

The frequency of the non-coding *SMAD3 *and *SMAD4 *variants from cases and controls was higher than values reported by Cargill *et al*., Halushka *et al*. when considering the value reported for the European-American samples, and the 3'UTR region reported by Ten Asbroek *et al*. (Table 3). However, this may be simply due to the difference in the study design, where up to 150 bp of the non-coding exon-intron boundaries were covered in this study compared to < 18 bp in the reference studies.

### *In silico *analyses indicate potential mechanisms of inactivation

Detailed results for bioinformatic analyses of ASSA and FastSNP are summarized in Additional file 1, Table S3. Among 11 intronic variants in *SMAD3*, only IVS8-55A > G, identified in two population controls was predicted to abolish a branch site (Table 2). Four of the 16 identified SMAD4 variants were predicted to create cryptic sites (Table 2). The *SMAD4 *variants, c.1350G > A (p.Gln450Gln) (P9) found in a patient with familial breast cancer and c.1214T > C (p.Phe362Phe) from a familial breast cancer case and a control, were predicted to result in the disruption of exonic splicing enhancers (ESE).

### Altered expression but no cryptic site formation in breast cancers

In addition to the seven potential mutations predicted to be functional, all the rarely occurring variants in our population were assessed by RT-PCR for a thorough investigation of both the effect of the predicted splicing mutants and alterations on intronic structures such as ISS/ISE, which presently cannot be reliably predicted *in silico *and might otherwise be missed. However, the gel electrophoresis of the PCR products showed the absence of aberrantly spliced transcripts in the mRNA panel studied.

For the qPCR analysis, cDNA harboring *SMAD3 *and *SMAD4 *variants were categorized as breast cancer cases with variants (BC-VAR) and controls with variants (CO-VAR). As the case/control samples with *SMAD3 *variants were negative for *SMAD4 *variants and vice versa, each was used as a negative control for the other to increase the power of the analysis (denoted BC-REF, CO-REF).

**Figure 1:**
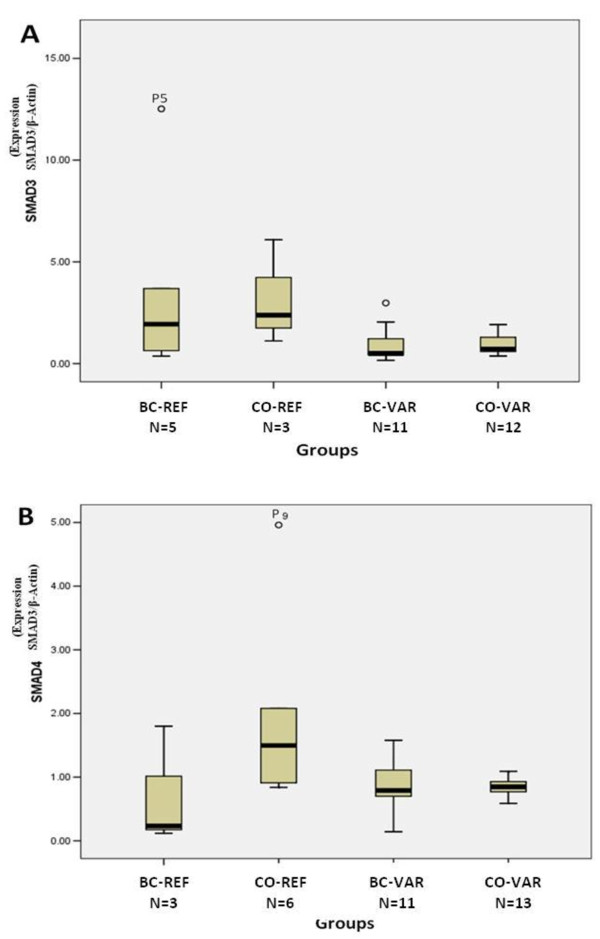
**Quantitative Real-Time PCR analysis of *SMAD3 *and *SMAD4 *germline expressions**. The mean expressions of four separate groups of mRNA are compared for (**A**) SMAD3 and (**B**) SMAD4. The BC-VAR (Breast Cancer-Variants) group represents cases harboring variants; the BC-REF (Breast Cancer - Reference) group represents the cases where variants were not detected. The CO-VAR (Control-Variants) group represents controls harboring variants; CO-REF (Control-Reference), represents controls where variants were not detected. P5 and P9 represent familial breast cancer cases exhibiting high germline expression with the latter harboring the novel c.1350G > A alteration. Statistical significance was determined by the Mann-Whitney test of independence with error bars representing standard deviation (SD). The upper and lower boundaries of the box indicate 75th and 25th percentiles, respectively. The line within the box represents the median; bars above and below the box, the 90th and 10th percentiles, respectively.

*SMAD3 *expression levels in breast cancers harboring variants (BC-VAR; *n *= 3) were significantly higher compared to CO-VAR (*n *= 11; *P *= 0.038) and CO-REF (*n *= 12; *P *= 0.035) (Figure 1a). However, the *SMAD3 *variants of the MH2 domain presented here do not seem to be a strong driving force for the observed change in expression. IVS8+23A > C was found in two cases (P1, P8) with a large six-fold expression increase in P8 but unchanged in P1 (Table 4; Additional file 1, Table S4a) and IVS9+132A > T from P4 showing a 2.39-fold increase (BC-VAR group mean: 3.19 ± 0.78). Particularly, P5 in *SMAD3 *BC-REF (*n *= 5) had a very high increase in expression (> 12-fold) without the presence of an MH2 variant (BC-REF group mean 3.83 ± 0.78). Nevertheless, significantly higher mean expression levels in the grouped breast cancer (BC) versus control (CO) (*P *= 0.02) (data not shown), strongly suggests *SMAD3 *germline expression to be an important factor in breast cancer.

**Table 4:** Clinical characteristics of 37 cases or controls with germline variants

			Variant Detected	
Patient ID	Age	Familial*	SMAD3	SMAD4
P1	53	OFBCR	IVS8+23A > C	None
P2	57		None	IVS12+41 G > A
P3	39	OFBCR	None	IVS10+109 A > G
P4	45	OFBCR/FDR	IVS9+132 A > T	None
P5	44		None	IVS11+118 A > G
P6	51	OFBCR	None	IVS12-33 T > A
P7	47		None	IVS12 +132 delA
P8	N/A	OFBCR/FDR	IVS8+23A > C	None
P9	N/A	OFBCR	None	c.1350G > A/p.Gln450Gln
C1	44		None	IVS10+44 T > C, IVS11+106del7N
C2	44		None	IVS11+68 delGAA
C3	42		IVS8+23A > C	None
C4	37		None	IVS10+109 A > G
C5	49		None	IVS12 +132 delA
C6	34		IVS8-211 C > T	None
C7	43		None	IVS12-52 A > T
C8	43	OFBCR/FDR	IVS8+23A > C	None
C9	54		IVS8+23A > C	None
C10	45		IVS6-132C > T	None
C11	50		IVS7+69G > C	None
C12	52		None	c.1214T > C/p.Phe362Phe **
C13	47		None	IVS9-121 A > C
C14	50		IVS8+161C > T	IVS10+109 A > G
C15	31	OFBCR/FDR	none	IVS10+109 A > G
C16	35		IVS8+23A > C	None
C17	48		IVS8+23A > C	None
C18	46		IVS8+48T > G	None
C19	43		IVS7+69G > C, IVS8-55 A > G	None
C20	46		None	IVS13+53 A > G
C21	45		IVS8-170C > T	None
C22	67		IVS8+48T > G	None
C23	49		None	IVS10-121 A > C
C24	37		None	c.1701A > G/p.Ile525Val
C25	52		IVS7+69G > C, IVS8-55 A > G	None
C26	66		IVS7+69G > C	None
C27	60		None	IVS12 +132 delA
C28	62		None	IVS12 +132 delA

Note: P represents a patient and C represents control

* The familial categorization is based on the OFBCR (Ontario Familial Breast Cancer Registry) and/or FDR (First Degree Relative) which indicates the strength of familial association based on clinical history

** Found also in a case but mRNA was unavailable from the Breast Cancer Family Registry repository

The *SMAD4 *variants predicted to create cryptic sites or abolish branch sites did not result in aberrant expression patterns, consistent with the RT-PCR results. However, BC-VAR, but not BC-REF, exhibited significant up-regulation in expression relative to CO-REF (*n *= 11; *P *= 0.036), CO-VAR (*n *= 13; *P *= 0.037) (Figure 1b; Additional file 1, Table S4b). Among the BC-VAR group (mean: 1.96 ± 0.42) were P2 (IVS12 + 41G > A) and P5 (IVS11 + 118A > G), which showed a moderate two-fold increase in expression. Of note, P9 harboring c.1350G > A (p.Gln450Gln) from a familial breast cancer case (Table 4) predicted to disrupt ESE motifs was associated with a level of high expression (> 5-fold) that was not seen in any of the sample studied.

### Somatic mutations in *SMAD3 *and *SMAD4*

According to COSMIC, no *SMAD3 *mutations were reported based on 48 breast tumors screened while two *SMAD3 *homozygous mutations were identified from 38 colorectal tumors. *SMAD4 *somatic mutations were clustered in the MH2 domain supporting the observation that the MH2 domain is a mutation hotspot in many cancer types. For breast cancer the four homozygous whole gene deletions represented the 2.8% of mutations identified from the screening of 141 tumor samples, while 10.7% (92/858) and 21.8% (123/564) were tumorigenic mutations of the large intestines and pancreas, respectively.

### Expressions of *SMAD3 *and *SMAD4 *are up-regulated in breast carcinoma

**Figure 2:**
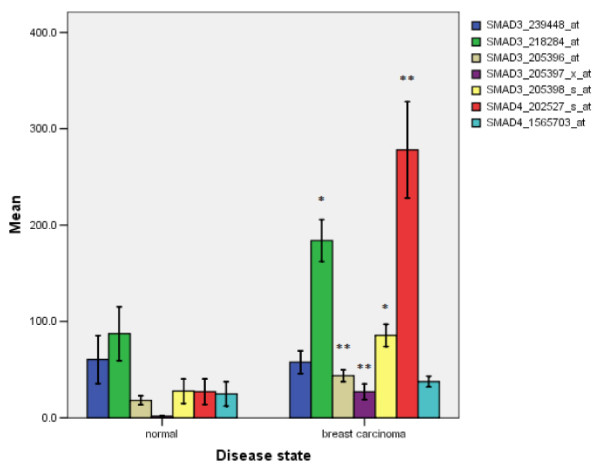
**SMAD3 and SMAD4 expression in breast carcinoma relative to normal tissue**. Expression data were obtained from Affymetric GeneChip U133 Plus 2.0 arrays for 50 tumors and 10 surrounding unaffected tissues. Statistical significance was determined by two independent sample t-tests and Levin's tests for the equality of variance.

Using publically available online tissue expression data [40], *SMAD3 *and *SMAD4 *expression in breast tumors versus normal breast tissue were assessed using two independent samples t-test and Levin's test for the equality of variance. SMAD3 and SMAD4 mRNA expression levels were found to be significantly elevated in the tumor tissues compared to normal tissues for four of five probes (> 5-fold average increase, *P *< 0.05) and one of two probes (> 10-fold increase, *P *< 0.01) (Figure 2).

## Discussion

*BRCA1 *and *BRCA2 *are the most prominent breast cancer susceptibility genes. However, there remains a need to identify additional susceptibility genes as it has become increasingly evident that *BRCA1/BRCA2 *mutations cannot explain all cases of familial breast cancer. Two candidate genes that are of potential interest in clinical genetics of breast cancer are *SMAD3 *and *SMAD4*, encoding the key signaling transduction proteins of the Transforming Growth Factor-β (TGF-β) pathway. The *loci *on which they reside are frequently lost in breast cancer but whether germline variants are playing a role in predisposition of breast cancer has not been studied.

For the discovery of the variants we applied the dHPLC methodology, complemented by direct sequencing, which has been reported to have over 95% sensitivity and accuracy in detecting genetic variations [50]. We have targeted the analysis of the functionally critical MH2 domain because it has been shown to be a mutational hot spot in *SMAD4 *[29], the region where the putative *SMAD3 *mutations had been identified [15,16] and the region that interacts with BRCA1 [12]. Thus we reasoned that a comprehensive screen of the exons encoding the MH2 domain and surrounding intronic region represents the most effective design to detect novel *SMAD3 *and *SMAD4 *mutations.

Based on current understanding, mutations in *SMAD3 *are absent in almost all cancer types while mutations of *SMAD4 *are frequent in pancreatic and colorectal cancers but rare in breast cancer. However, it has been difficult to ascertain whether *SMAD3 *and *SMAD4 *mutations in breast cancer are truly rare or this understanding is due to the comparatively small sample sizes screened as noted from COSMIC. Furthermore, whether inactivating germline mutations are playing a role in breast cancer susceptibility has not yet been investigated.

Our analysis did not detect coding variants in the MH2 domain of *SMAD3*. In *SMAD4 *we identified two novel coding variants c.1350G > A (p.Gln450Gln) (P9), and c.1701A > G (p.Ile525Val) (C24) in a breast cancer case and control population, respectively, in addition to the previously known c.1214T > C (Phe362Phe) (rs1801250). As it has been suggested that SMAD3 and SMAD4 mutations are rare in breast cancer [14,26], we quantitatively assess whether this is the case in the germline. The identified variants were normalized relative to the base pairs screened and individuals assessed (θ) and our case-control results were compared to three large studies that have established an approximate frequency based on mutation analysis of germline DNA of healthy individuals representative of the natural rate of mutation.

We found that the nucleotide diversity in both cases and controls in the coding region of *SMAD3 *to be far less than all three reference studies. This difference is not attributable to a discrepancy in sensitivity of detection of germline variants since there were comparable frequencies for non-coding variants for both *SMAD3 *and *SMAD4*. This strongly supports that *SMAD3 *alteration is very infrequent and suggests that the MH2 domain is under stringent selective pressure where deleterious mutations impeding proper function could also negatively influence tumorigenesis. Within the coding region of *SMAD4*, on the other hand, nucleotide diversity estimations indicated that variants in cases and controls appear to occur at a similar, albeit lower, rate than the reference samples. This demonstrates that *SMAD4 *is not preferentially mutated in the breast, though rare genetic alterations may exist in the MH2 coding region.

The non-coding regions have a higher θ compared to the reference studies. However, it should be noted that both the Cargill *et al*. and Halushka *et al*. studies remarked that their non-coding regions are comprised of perigenic sequences (< 18 bp from the exon) while our study spans up to 150 bp of the intron and may be more representative of a neutral rate of polymorphism. In fact, the study by Cargill *et al*. suggest that the θ for four-fold degenerate sites reported in their study had the highest nucleotide diversity (θ = 9.73 ± 2.46) and may approximate the neutral rate of polymorphism. If this θ is assumed to be the neutral rate of polymorphism then what was observed in the non-coding regions of *SMAD3 *and *SMAD4 *cases (θ = 13.24 ± 7.02, 7.56 ± 3.36, respectively) and controls (θ = 11.24 ± 4, 11.71 ± 4.17) would be in agreement.

Intronic variants, which constituted the major type identified in this study, are increasingly found to be associated with splicing defects (and ESE/ESS alterations) causing cancer among other disorders [51]. However, RT-PCR analysis has shown the absence of any aberrantly spliced transcripts, and no exon skipping was observable in any sample, including the novel *SMAD4 *c.1350G > A variant (P9). It is also possible that the aberrant transcripts are unstable and their degradation may have occurred during the blood processing. Although it is true that variants disrupting ESEs are associated with decreased splicing efficiency and/or splicing defect, there have been instances in which gain of function ESE mutation strengthens the enhancer element resulting in preferential exon inclusion. For example, most mutations of microtubule-associated protein tau (MAPT) that are associated with (frontotemporal dementia and Parkinsonism associated with chromosome 17 (FTDP17), a condition related to Alzheimer's disease, are translationally silent but increase splicing efficiency of exon 10 that increases the rate of inclusion through strengthening ESEs at the 5' end or weakening ESS at the 3' end [52]. In this regard the c.1350G > A variant may be prioritized for further studies. Based on these results it appears inactivating SMAD3 and SMAD4 germline mutations and splicing defects appear to occur very infrequently in breast cancer.

While the absence of inactivating MH2 germline mutations from this study provides compelling evidence that *SMAD3 *and *SMAD4 *mutations are truly rare in breast cancer, this study cannot comprehensively exclude the presence of other mutations since the Mad-Homology 1 (MH1) and the variable linker region were not screened. However, with respect to *SMAD3*, our screening did not detect coding variants, within the MH2 domain, including the ones previously identified in colon and pancreas. Given that the *SMAD3 *mutations are infrequent and that its expression is elevated in peripheral blood and tumor tissues, SMAD3 does not seem to be inactivated and is unlikely to contribute as a tumor suppressor during breast cancer development. With respect to SMAD4, 90% of all known somatic *SMAD4 *mutations reported are located in the MH2 domain, suggesting that the number of undetected mutations is expected to be low when analysis is confined to this mutation hotspot. This is also supported by mutation analysis conducted in JPS by Howe *et al*., [28] showing that in 77 patients, inactivating germline *SMAD4 *mutation were found in 18.2% (14/77) of the samples and of these, 16.9% (13/77) occurred in the MH2 domain. Similarly, mutation germline analysis by Pyatt *et al*., [27] showed that *SMAD4 *is mutated in 18.6% (13/70) of the 70 JPS patients screened and of these, 12.7% (9/70) occurred in the MH2 domain. Lastly, a mutation screen of 56 patient thyroid tumor samples by Lazzereschi *et al*. 2005 [53] identified *SMAD4 *MH1 mutations as well as linker mutations leading to splicing defects. Nevertheless, the authors also found that more than half (53% (8/15)) of the mutations were missense mutations in the MH2 domain. By contrast, our study of 408 patient samples and nucleotide diversity analysis both show that inactivating MH2 domain mutations appear to be absent. Thus, by inference the remaining part of the gene is expected to harbor only very rare mutations. It should be noted that germline biallelic inactivations were not addressed in this study. For *SMAD4*, homozygous deletion mutations have been identified in invasive ductal carcinomas and it still remains a possibility that biallelic inactivation due to germline homozygous deletions could be playing a significant role in tumorigenesis. This possibility is currently under investigation.

Gene expression in peripheral blood cells has been shown to be altered in early breast cancer but not healthy controls [54,55]. To determine whether any of the variants are associated with altered expression levels we also performed expression analysis in the same sample set. Interpreting how changes in expression of *SMAD3 *and *SMAD4 *affect their activities in the cell may distinguish their roles as a tumor suppressor or oncogene in breast cancer susceptibility.

There is strong evidence for tumor suppressor function of SMAD3 as its loss is associated with tumorigenesis in various cancers [8-10]. However, our qPCR analysis showed that mRNA from breast cancer cases was significantly highly expressed relative to both control groups (BC vs. CO; *P *< 0.05, t-test) but was not due to the variants found in the breast cancer cases. Thus, this observation is likely attributable to regulatory factors beyond the MH2 domain. These results, together with the lack of inactivating mutations from this study and COSMIC database, strongly support that SMAD3 is not functioning as a direct tumor suppressor in breast cancer. Nevertheless the abnormally high levels of germline expression as well as statistically significant over-expression of *SMAD3 *in invasive ductal carcinoma (IDC) compared to normal tissues raises the possibility that epistatic interactions of SMAD3 may contribute to the oncogenic activities of TGF-ß. SMAD3 has been shown to counteract BRCA1-dependent DNA repair in response to DNA damaging agents and over-expression of SMAD3 decreases BRCA1-dependent cell survival [12]. Therefore, it is possible that such high levels of germline *SMAD3 *expression may mimic a BRCA1-deficient phenotype. Furthermore, the aberrant expression may be a mechanism that reconciles the allelic imbalance often associated with the 15q21 locus in breast cancer [11] with the apparent lack of *SMAD3 *inactivating mutations.

Loss of expression and allelic imbalance at the *SMAD4 *locus has been shown to promote carcinogenesis of gastric, ovarian, and colorectal cancers [18,47,48]. Overall, in our study SMAD4 cases were not differently expressed compared to controls and the variants predicted to create cryptic sites or abolish branch site did not result in aberrant expression. Interestingly, however, the breast cancer case (P9) harboring the novel c.1350G > A variant in exon 10 of *SMAD4*, predicted to affect ESEs, had a significant expression increase by almost five-fold that was not observed in any other samples examined, indicating that the full length transcript is preferentially over-produced. Increasing SMAD4 germline expression is unlikely to predispose to breast cancer due to its important role as a tumor suppressor suggesting that SMAD4 is not involved in susceptibility. However, it is appreciated that as tumorigenesis develops the cell becomes increasingly desensitized to the anti-proliferative effects of TGF-β but remains susceptible to its oncogenic properties. Therefore, c.1350G > A could represent a potential prognostic marker as SMAD4 expression has been shown to be an important mediator in the development of osteolytic bone metastasis in late cancer stages but is not required in its maintenance or progression [56,57]. This is consistent with the fact that although SMAD4 mRNA levels and protein expression appear to be decreased in breast cancer relative to normal tissues [58] they are not significantly correlated with tumor size, metastases, nodal status, histological grade, histological type, or estrogen receptor expression. In fact, there was a trend toward longer survival times in patients with SMAD4 negative tumors [58] and a loss of expression is also correlated with a decrease in axillary lymph node metastasis [59]. Thus, the results presented here highlight a potential value for evaluating coding variants that affect ESE/ESS for abnormal expression even if they do not influence splicing.

## Conclusions

This study has demonstrated that expression levels of SMAD3 and SMAD4 are important factors in breast cancer but have different consequences. Aberrant *SMAD3 *germline expression rather than inactivating mutations may be playing a major role in susceptibility. While *SMAD4 *is not preferentially mutated in breast cancer, rarely occurring variants, such as c.1350G > A, probably serve as low to medium penetrating inherited germline mutations with prognostic value. Despite the fact that such expression-altering variants only account for a small subset of the alterations observed, their importance should not be underestimated since it is possible that human cancers can possess many low-penetrating alterations which, when acting synergistically, represent a powerful driving force of the carcinogenic process. Future research to explore the mechanisms of deregulation of SMAD3 and SMAD4 expressions will be essential in determining their association with breast cancer risk and tumorigenesis.

## Abbreviations

ASSA: Automated Splice-Site Analyses; Breast CFR: Breast Cancer Family Registry; COSMIC: Catalogue of Somatic Mutations in Cancer; DHPLC: Denaturing High Performance Liquid Chromatography; ESE: Exonic Splicing Enhancer; ESS: Exonic Splicing Silencer; FastSNP: function analysis and selection tool for single nucleotide polymorphisms; MH2: Mad-Homology 2; PolyPhen: Polymorphism Phenotyping; RT-PCR: reverse-transcription PCR; QPCR: quantitative real-time PCR; SIFT: sort intolerant from tolerant; SNP: single nucleotide polymorphism; TGF-β: transforming growth factor β

## Competing interests

The authors declare that they have no competing interests.

## Authors' contributions

ET contributed to study design and led the mutation screening and data analysis, and drafted the manuscript. II contributed to data analysis, statistical analyses and helped to draft the manuscript. LB contributed to data analysis and statistical analyses, while JK was responsible for subjects ascertained through the Breast Cancer Family Registry, and helped to revise the manuscript. IL was responsible for subjects ascertained through the Breast Cancer Family Registry and helped to revise the manuscript. HO contributed to study design, the data analysis, and drafting of the manuscript. All authors have read and approved the final version of the manuscript.

## Supplementary Material

Additional file 1 **Supplemental materials**. Additional information on experimental conditions and detailed description of data. Click here for file
