# Supplementary material for: Identification of germline alterations of the mad homology 2 domain of SMAD3 and SMAD4 from the Ontario site of the breast cancer family registry (CFR)
Source: Breast Cancer Res. 2011 Aug 11;13(4):R77. doi: 10.1186/bcr2926 (PMC3236341; doi:10.1186/bcr2926)
Supplement: Additional file 1 — Supplemental materials. Additional information on experimental conditions and detailed description of data. [file bcr2926-S1.PDF]

SUPPLEMENTARY DATA

Table S1: Reverse Transcription-PCR (RT-PCR) Conditions

Gene	Exons	Amplicon size (bp)	Annealing Temperature (°C)	Mg2+ (mM)	Forward PCR Primer (5'-3')	Reverse PCR Primer (5'-3')
SMAD3	6 to 9	688	61	1.5	TAGGGCTGCTCTCCAATGTCAA	TGGGGCCAAAGGGTAAATGTGT
SMAD4	8 to 11	519	56	1.5	TATAAATGAGGCTGGTCTAGGG	GACATGCCTACTACGACCGTAG

Table S2a: Sample characteristics of nucleotide diversity studies

	Base Pairs Screened (bp)				
**Site of Variation**	**This Study (SMAD3)**	**This Study (SMAD4)**	**Cargill et al**	**Halushka et al**	**Ten Ashbroek et al**
**Non-Coding**	1135	1090	60,410	102607 *	8884 **
**Coding**	415	688	138,823	86,946	42,270
Total	1550	1778	196,233	189,553	51,154
Characteristic of Study Genes	Signaling	Signaling	cardiovascualr, endocrine, neurological systems genes	Blood-Pressure genes	Highly Conserved essential genes
**Total # Individuals Screened**	408 Cases 710 Contr	408 Cases 710 Contr	57 (20 european, 14 asian, 10 African american, 7 african pygmies, 10 european)	74 (40 zimbawe, 32 Michigan, 3 N. European)	36 (4 chinese, 4 japanese, 2 oterh asians, 1 india, 1 saudi arabia, 4 african american, 4 hispanic american, 5 white of south european origin, 11 whites of central/eur origin)

* Including 5' and 3' UTR
** 3' UTR analyzed

Table S2b: Polymorphism counts for nucleotide diversity studies

	# Polymorphisms detected				
	This Study (SMAD3)	This Study (SMAD4)	Cargill et al	Halushka et al	Ten Ashbroek et al
**Non-Coding**	10 (Control) 4 (Case)	10 (Control) 6 (Case)	168	487 *	N/A
**Coding**	0 (Control) 0 (Case)	2 (Control) 2 (Case)	392	387	65
Synonymous	0 (Control) 0 (Case)	1 (Control) 1 (Case)	207	178	N/A
Non-synonymous	0 (Control) 0 (Case)	1 (Control) 1 (Case)	185	209	N/A
**Total**	10 (Control) 4 (Case)	12 (Control) 8 (Case)	560	874	65

* 150 variants were intronic, the remaining were found in the 3' UTR

Table S3: Detailed Bioinformatic output for biologically relevant variants

SMAD3 (MH2)	Genetic Variant	Case or Control	ASSA (WT > Mut)	Predicted Outcome
**Exon 9**	IVS8-55 A>G	Control	Branch Point Change 2.6 > -9.3 (-453.8%)	Abolished branch point

SMAD4(MH2)	Genetic Variant	Case or Control	ASSA (WT > Mut)	Predicted Outcome
**Exon 10**	IVS10+109 A>G	Cases and Control	Cryptic donor -9.1 > 3.7 (+345.9%)	Creation of a cryptic donor site 108 bp upstream of wildtype donor
**Exon 11**	IVS11+126 delTATATTA	Control	Cryptic branch point -7.7 > 3.8 (+302.6%)	Creation of a cryptic branch site 113 bp upstream of wildtype donor
**Exon 12**	IVS12-52 A>T	Control	Cryptic donor -4.5 > 3.3 (+236%)	Creation of a cryptic donor site 53 bp upstream of wildtype donor site
	IVS12-33 T>A	Case	Cryptic branch point -4 > 7.9 (+297.5%)	Creation of a cryptic branch site 32 bp upstream of wildtype acceptor

SMAD4(MH2)	Genetic Variant	Case or Control	Evolutionary Conservation		FASTsnp	
			**SIFT**	**PolyPhen**	**ESE-Finder**	**Predicted Outcome**
**Exon 10**	c.1214T>C / p.Phe362Phe	Cases and Control	NA	NA	Loss SC35 & Srp55	Loss of ESE Motifs
**Exon 12**	c.1478G>A / p.Asp450Asp	Case	NA	NA	Gain Srp55: 3.3435 Loss SF2/ASF and SC35	Loss/Gain of ESE Motifs
**Exon 13**	c.1701A>G / p.Ile525Val	Control	Tolerated	Benign	None Found	No Changes to Splicing Pattern

Table S4a: Detailed real-time PCR results for SMAD3 groups

Patient ID	Age	Familial	SMAD3 Variant(s)	SMAD3/B2M Ratio	Classification	Mean ± SD
P3	39	OFBCR		0.36 ± 0.02	BC-REF	
P6	51	OFBCR		0.64 ± 0.01	BC-REF	
P7	47			1.94 ± 0.1	BC-REF	3.83±0.78
P2	57			3.69 ± 0.1	BC-REF	
P5	44			12.52± 1.8	BC-REF	
P1	53	OFBCR	IVS8+23A>C	1.11 ± 0.08	BC-VAR	
P4	45	OFBCR/FDR	IVS9+132 A>T	2.39 ± 0.1	BC-VAR	3.19±0.35
P8	N/A	OFBCR/FDR	IVS8+23A>C	6.08 ± 0.7	BC-VAR	
C13	47			0.15±0.002	CO-REF	
C7	43			0.43±0.03	CO-REF	
C5	49			0.47±0..03	CO-REF	
C24	37			0.48±0.02	CO-REF	
C28	62			0.49±0.03	CO-REF	
C23	49			0.5±0.07	CO-REF	0.93±0.066
C1	44			0.83±0.1	CO-REF	
C4	37			0.84±0.05	CO-REF	
C15	31	OFBCR/FDR		1.61±0.05	CO-REF	
C27	60			2.05±0.2	CO-REF	
C2	44			2.98±0.2	CO-REF	
C22	67		IVS8+48T>G	0.35±0.05	CO-VAR	
C14	50		IVS8+161C>T	0.37±0.008	CO-VAR	
C10	45		IVS6-132C>T	0.42±0.03	CO-VAR	
C9	54		IVS8+23A>C	0.59±0.05	CO-VAR	
C25	52		IVS7+69G>C, IVS8-55 A>G	0.60±0.2	CO-VAR	
C18	46		IVS8+48T>G	0.62±0.1	CO-VAR	2.03±0.071
C26	66		IVS7+69G>C	0.7±0.07	CO-VAR	
C3	42		IVS8+23A>C	0.73±0.07	CO-VAR	
C6	34		IVS8-211 C>T	0.91±0.05	CO-VAR	
C19	43		IVS7+69G>C, IVS8-55 A>G	1.68±0.1	CO-VAR	
C8	43	OFBCR/FDR	IVS8+23A>C	1.82±0.2	CO-VAR	
C17	48		IVS8+23A>C	1.92±0.2	CO-VAR	
C11	50		IVS7+69G>C, IVS8-55 A>G	N/A	Removed	
C20	46			N/A	Removed	N/A
C21	45		IVS8-170C>T	N/A	Removed	
C12	52			ND	ND	
C16	35		IVS8+23A>C	ND	ND	N/A
P9	N/A	OFBCR		ND	ND	

Removed: Samples removed due to low mRNA quality

ND: Not done due to limitation in template cDNA

Table S4b: Detailed real-time PCR results for SMAD4 groups

Patient ID	Age	Familial	SMAD4 Variant(s)	SMAD4/B2M Ratio	Classification	Mean ± SD
P4	45	OFBCR/FDR		0.12±0.02	BC-REF	
P8	N/A	OFBCR/FDR		1.8±0.3	BC-REF	0.72±0.17
P1	53	OFBCR		0.23±0.01	BC-REF	
P7	47		IVS10 +132 delA	0.84±0.03	BC-VAR	
P6	51	OFBCR	IVS10-33 T>A	0.91±0.09	BC-VAR	
P3	39	OFBCR	IVS8+109 A>G	0.97±0.01	BC-VAR	1.96±0.42
P5	44		IVS9+118 A>G	2.02±0.2	BC-VAR	
P2	57		IVS10+41 G>A	2.08±0.5	BC-VAR	
P9	N/A	OFBCR	c.1478G>A / p.Asp450Asp	4.96±1.1	BC-VAR	
C22	67			0.14±0.01	CO-REF	
C3	42			0.67±0.02	CO-REF	
C18	46			0.68±0.06	CO-REF	
C17	48			0.72±0.01	CO-REF	
C19	43			0.77±0.1	CO-REF	
C9	54			0.79±0.04	CO-REF	1.08±0.1
C25	52			0.8±0.05	CO-REF	
C16	35			1.1±0.09	CO-REF	
C8	43	OFBCR/FDR		1.12±0.1	CO-REF	
C6	34			1.19±0.2	CO-REF	
C10	45			1.58±0.1	CO-REF	
C28	62		IVS10 +132 delA	0.59±0.06	CO-VAR	
C23	49		IVS8-121 A>C	0.67±0.1	CO-VAR	
C7	43		IVS10-62 A>T	0.69±0.06	CO-VAR	
C12	52		c.1214T>C / p.Phe362Phe	0.77±0.1	CO-VAR	
C24	37		c.1701A>G / p.Ile525Val	0.78±0.09	CO-VAR	
C14	50		IVS8+109 A>G	0.8±0.08	CO-VAR	
C13	47		IVS7-121 A>C	0.85±0.08	CO-VAR	0.85±0.048
C1	44		IVS8+44 T>C	0.87±0.2	CO-VAR	
C5	49		IVS10 +132 delA	0.92±0.07	CO-VAR	
C15	31	OFBCR/FDR	IVS8+109 A>G	0.93±0.1	CO-VAR	
C2	44		IVS9+68 delGAA	1.06±0.09	CO-VAR	
C27	60		IVS10 +132 delA	1.07±0.05	CO-VAR	
C4	37		IVS8+109 A>G	1.09±0.2	CO-VAR	
C11	50			N/A	Removed	
C20	46		IVS11+53 A>G	N/A	Removed	N/A
C21	45			N/A	Removed	
C26	66			ND	ND	N/A

Removed: Samples removed due to low mRNA quality

ND: Not done due to limitation in template cDNA
